# Using Trust to Secure Geographic and Energy Aware Routing against Multiple Attacks

**DOI:** 10.1371/journal.pone.0077488

**Published:** 2013-10-21

**Authors:** Guanghua Zhang, Yuqing Zhang, Zhenguo Chen

**Affiliations:** 1 Key Lab of Computer Networks and Information Security of Ministry of Education, Xidian University, Xi’an, Shaanxi, China; 2 College of Information Science and Engineering, Hebei University of Science and Technology, Shijiazhuang, Hebei, China; 3 National Computer Network Intrusion Protection Center, Beijing, China; 4 School of Information Science & Engineering,Northeastern University,Shenyang, Liaoning, China; 5 Department of Computer Science and Technology, North China Institute of Science and Technology, East Yanjiao, Beijing, China; Semmelweis University, Hungary

## Abstract

To address the vulnerability of geographic routing to multiple security threats such as false routing information, selective forwarding and the Sybil attack in wireless sensor networks, this paper proposes a trust-based defending model against above-mentioned multiple attacks. Considering the characteristics of resource-constrained sensor nodes, trust values of neighboring nodes on the routing path can be calculated through the Dirichlet distribution function, which is based on data packets' acknowledgements in a certain period instead of energy-consuming monitoring. Trust is combined with the cost of geographic and energy aware routing for selecting the next hop of routing. At the same time, the initial trust is dynamically determined, service requests are restricted for malicious nodes in accordance with trust values, and the impact of node mobility is weakened by the trust evolution. The simulation results and analysis show that the proposed model under multiple attacks has advantages in packet delivery ratio and network lifetime over the existing models.

## Introduction

In recent years, wireless sensor networks (WSNs) have been widely developed and applied in environmental monitoring, smart home, medical health, etc. [1]. WSNs are self-organized dynamic multi-hop network systems, which consist of many low-cost micro-sensor nodes communicating with each other through wireless channels. As a key technology in WSNs, routing protocols are responsible for reliable transmission of data packets among sensor nodes [2], including two basic functions of finding the optimal path from the source node to the destination node and transmitting data packets along the optimal path. Based on constraints of computing, communication, storage, energy of nodes and requirements of various applications, there have been flat routing protocols [3], hierarchical routing protocols [4], query-based routing protocols [5], geographic routing protocols [6], etc. During the design these routing protocols are required to improve network performance as much as possible, but most of them do not take into account the routing security issues.

Routing protocols for wireless sensor networks are often subject to the following types of attacks [7]: false routing information, selective forwarding, the Sybil attack, sinkhole attacks, wormholes, HELLO flood attacks, and acknowledgement spoofing. In all scenarios where various routing protocols are applied, there are not always serious security threats and all the routing attacks. Literature [7] points out that geographic routing protocols are more promising, in which location information is used for routing selection and data packets' transmission by mutual cooperation of intermediate nodes according to certain strategies. However, under certain conditions, some intermediate nodes may be no longer reliable due to being captured or other reasons, which makes geographic routing protocols vulnerable to false routing information, selective forwarding attack, and the Sybil attack.

Traditional password-based security systems are mainly used to defend against external attacks so that they usually cannot resolve the internal attacks resulting from nodes being captured. In P2P and ad hoc networks trust management has been extensively studied [8], [9] and can be considered as an effective approach to defend against internal attacks in wireless sensor networks. Trust management is the behavior of trust evaluation through collection, analysis and coding of relevant evidence. Along with the development of distributed applications, many researchers have gradually realized the security function of trust mechanism, using trust to ensure reliable interaction between distributed entities. In geographical and energy aware routing (GEAR) protocol [6] for WSNs, attackers can launch the Sybil attack by faking location information. At the same time, attackers continue to broadcast that they have the maximum energy level, and induce the surrounding nodes to choose them as the next hop. These make it easy for attackers to put themselves in data transmission path and implement selective forwarding attack. Therefore, the security challenges for GEAR protocol are put forward. To defend against malicious routing behavior from combination of the above three attacks, trust mechanism is introduced to perform trust evaluation based on nodes' routing behaviors for identifying malicious nodes and enhancing the robustness of GEAR. The rest of this paper is organized as follows: Section 2 discusses related work. Section 3 presents a trust-based defending model for GEAR—TBDM-GR. This is followed by simulation analysis and advantages over the existing models in Section 4, and Section 5 concludes the paper.

### Related work

In a typical representative of geographic routing protocols-GEAR [6], each node knows its own location and remaining energy level, and learns about all its neighbors' locations and remaining energy levels through a simple HELLO message exchange mechanism. Based on the location of the target region and the nodes' energy levels, the optimal path from the convergence node to the target region is established to avoid flooding queries and reduce the routing cost.

The security threats to which GEAR is vulnerable are deeply analyzed in literature [7]. The GEAR protocol relies on nodes' locations, which makes it easy for attackers to forge the locations and appear in different coordinates at the same time for launching the Sybil attack regardless of their actual locations. Meantime, the GEAR protocol considers nodes' remaining energy level as a factor for routing selection. Attackers continue to broadcast that they have the maximum energy level, and induce the surrounding nodes to choose them as the next hop. When an attacker gets routing tasks in any of the above-mentioned ways, it may perform selective forwarding—it refuses to forward particular data packets or directly drops them. Recently trust management has been applied for secure routing in literatures [10]–[18].

In literature [10], the watchdog mechanism is used to continuously monitor the forwarding behaviors of neighbors and report observations to the rating component. In literature [11], a method of periodically monitoring neighboring nodes at a certain probability is used to save energy while ensuring the system performance. These two methods both need a cache. If a neighboring node timely forwards data packets which are the same as those in the local cache, the neighboring node can be regarded as reliable and the trust value is increased. Otherwise, the trust value of the node is reduced. However, even with the monitoring technology in WSNs, it is also difficult to judge whether a node has correctly forwarded a received data packet [7] because a malicious node can forward the data packet to a node that is not on the path, and even forward it to other malicious nodes, causing routing loops. In this paper, the forwarding behaviors of neighboring nodes are evaluated by acknowledging data packets. Therefore, energy consumption in the monitoring is reduced and the network lifetime is prolonged.

In literatures [12]–[14], the forwarding behaviors of nodes are divided into two states: success or failure, and the number of successful and failed forwarding behaviors is counted to calculate the nodes' trust values. However, the exact definition of success or failure in forwarding is not provided. At the same time, nodes' routing behaviors present imprecision and uncertainty under the influence of the quality of wireless channels and node malfunction. Therefore, the traditional trust calculation method based on binary logic will be no longer suitable for WSN. In trust calculation, we consider the impact of non-malicious factors such as the quality of wireless channels, node malfunction, etc. Neighboring nodes' trust values are calculated by the Dirichlet distribution based on counting the number of successful, uncertain, and failed forwarding behaviors weighted by the importance of data packets.

In literature [11], when trust-based routing selection is carried out, the trust and the calculated cost in the GEAR protocol are respectively given a certain weight, and this method assumes full compensability between trust and cost. That is to say, no matter how poor an attribute of a node is, it can be compensated by other attributes. The cost to trust ratio is calculated in literature [14], and the trust to cost ratio is calculated in literature [15]. These two schemes all lack of theoretical and experimental supports to some extent. In this paper, the multi-attribute decision making method is used to take advantage of trust as objectively as possible in trust-based routing selection and to balance the relationship between trust and cost.

In addition, there have been some defending strategies against false routing information, selective forwarding and the Sybil attack. For instance, the received signal strength in [16] and physical layer network coding in [17] are used to defend against the Sybil attack, and the multi-path routing in [18] is used to defend against selective forwarding. These solutions are designed with the purpose of solving some specific attack instead of multiple attacks that are jointed generally to imperil GEAR. In this paper, the trust-based defense against multiple attacks is implemented by means of dynamic initial trust, the incentive mechanism, etc., in combination with the specific characteristics of the GEAR protocol.

### Defending model based on trust

The trust-based defending model for GEAR—TBDM-GR is proposed in this section. Firstly, assumptions are given, followed by the trust calculation process. Finally, descriptions are given on how trust is combined with the cost in GEAR for routing selection, and how some defending strategies based on trust are deployed.

### Assumptions

GEAR [6] uses a geographic and energy-aware neighbor selection heuristic method to route packets towards the target region. Under most conditions, a recursive geographic forwarding algorithm is used to disseminate the packet within the region. However, under some low-density conditions, restricted flooding is adopted. Finally monitoring data is transmitted from the target region to the sink node. The proposed trust-based defending model does not change GEAR's original architecture, but it serves as a trust component supporting unicast data packet forwarding. In order to better focus on the defensive role of the model, we state the following assumptions.

First, a node has a unique identifier corresponding to its geographic location. If nodes launch the Sybil attack, different geographic locations have different identifiers.

Second, a secure communication channel is established with the help of the key management scheme [19] to protect trust information from traffic analysis or fabrication during transmission from one node to another.

Third, the IEEE 802.11 MAC protocol based on the distributed coordination function is introduced in the link layer. IEEE 802.11 keeps packets in their caches until the sender receives an ACK. Whenever the receiver successfully receives a packet, it will send an ACK to the sender. If the sender node does not receive an ACK for a packet during a predefined threshold period, it will retransmit that packet.

### Judgment on routing behaviors

After node i sends a data packet to its neighboring node j in one-hop transmission range, according to the third assumption, it should receive an acknowledgement from node j. Otherwise, node i will retransmit the data packet. Retransmission in the link layer is supposed to be caused by some non-malicious factors such as the quality of wireless channels, node malfunction, etc., and by attacks in the routing layer. For node *i*, the non-malicious impact factor 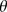 is calculated in Eq. (1) based on the retransmission rates of all its neighbors.
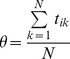
(1)


Where N represents the number of node *i*'s neighboring nodes, and for node *i* the retransmission rate of the neighboring node *k* within a certain period is denoted as 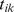, which is calculated by Eq. (2).
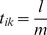
(2)


Where *l* represents the number of packets retransmitted from node *i* to node *k*, and *m* represents the total number of packets sent by node *i* to node *k*.

To judge a node's forwarding behaviors based on data packets' acknowledgements and calculate the neighboring nodes' trust values, we add the support for acknowledgements to the source node in the existing GEAR protocol. Therefore, nodes need not be in monitoring state for a long period, and the impact of a malicious node forwarding data packets on a node not on the routing path is eliminated to some extent. When node *i* forwards a data packet to neighboring node *j*, node *i* will record the information about this forwarding in its cache, where the information is denoted as *<source, destination, j, importance>*. “*source*” represents the source node of the data packet, “*destination*” represents the destination node of the data packet, “*j*” represents the next hop the data packet is forwarded to, and “*importance*” represents the importance of the data packet. During a predefined threshold period, if node *i* receives an acknowledgement from its neighboring node *j*, node *i* considers that the data packet has been successfully forwarded to the destination node through node *j*, and the number of successful forwarding times for node *j* is added by 1. Otherwise, the number of failed forwarding attempts for node *j* is added by 1. The acknowledgement is denoted as *<destination, source, j, importance>*. At the same time, node *i* records the data packets' importance for weighting the statistical results of the number of successful, uncertain and failed forwarding times, because a neighboring node that has forwarded data packets of greater importance is regarded to be more reliable, and vice versa. The weighted number of successful forwarding times is denoted as 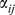. The weighted number of uncertain forwarding times is denoted as 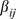. The weighted number of failed forwarding times is denoted as 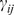. The three variables are calculated in Eqs. (3)∼(5).
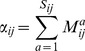
(3)

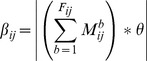
(4)

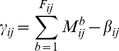
(5)


Where 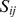 and 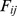
respectively represent the number of successful forwarding times and the number of failed forwarding times from node *i* to node *j*, and 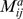 and 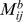
respectively represent the forwarded data packets' importance from node *i* to node *j*. 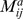 and 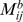 are in the range {1,2,3}, which respectively mean general, important and confidential data packets. The weighted number of uncertain forwarding times 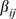 is defined as the rounding product of the weighted number of failed forwarding times and the non-malicious impact factor 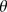, which is used to indicate the failed forwarding times caused by non-malicious factors.

### Trust calculation based on the Dirichlet distribution

In TBDM-GR, trust evaluation on nodes is also known as reliability evaluation, which is defined as the reliability of packets delivery to their intended next-hop. Reliability is determined by direct trust and indirect trust. Direct trust indicates statistics of results after a node directly interacts with its neighboring nodes. Indirect trust indicates information about the neighboring node provided by other neighboring nodes. The reliability evaluation of node *i* on neighboring node *j* is denoted as 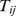.

#### 1) the principle of Dirichlet reputation system

In this paper, the Dirichlet distribution is used to compute 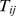 for the consideration of its flexibility, simplicity, simplicity, and great expression capacity [20]. The Dirichlet distribution is the equivalent of the Beta distribution [21] extended from binary events to multiple events, which can better give expression to routing behaviors in complex environment of WSNs. The Dirichlet distribution captures a sequence of observations of the *k* possible outcomes with *k* positive real parameters 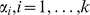, each corresponding to one of the possible outcomes. The probability density function of the Dirichlet distribution for variable vector 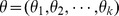 with parameter vector 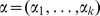 is given in Eq. (6).
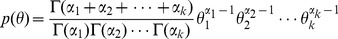
(6)


Where 
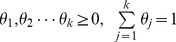
, and 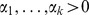. The operation 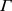 represents the Gamma function. The probability expectation of the Dirichlet distribution is shown in Eq. (7).
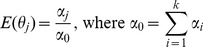
(7)


#### 2) the calculation of direct trust

According to Dirichlet reputation systems [20], the reputation is computed using Dirichlet density function of nodes' previous forwarding behaviors. Trust evaluation is the expected value of the reputation. Therefore 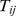 can be calculated in Eq. (8).
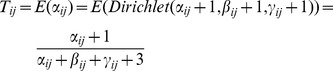
(8)


Where 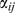,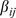 and 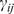 respectively represent the weighted number of successful forwarding times, the weighted number of uncertain forwarding times, and the weighted number of failed forwarding times, as described in Section 3.2.

In addition, the uncertainty measure 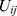 of node *i* on neighboring node *j* is introduced in Eq. (9). 
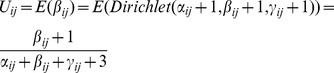
(9)


#### 3) the integration of indirect trust

Node *i*'s observations about node *j* is considered in the equation above, which involves only direct trust information. As a result, the system has a very large convergence time [22]. In our proposed model TBDM-GR, nodes can exchange information about their own neighboring nodes in order to obtain indirect trust information and integrate the information into trust evaluation. Node *i* has previous observations about node *j* denoted as 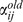, 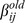 and 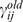. Furthermore, assume that in a period of 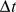, node *i* records new information about node *j*, denoted as 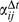, 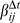 and 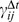. At the same time, node *i* receives indirect trust information about node *j* from a set of its neighboring nodes, which is denoted as 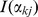, 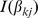 and 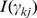. Then, the new weighted number of successful forwarding times, uncertain forwarding times, and failed forwarding times can be calculated in Eqs. (10)∼(12).
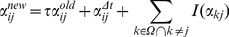
(10)

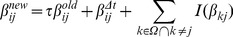
(11)

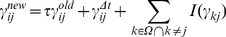
(12)


Where 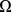 represents the set of node *i*’s neighboring nodes; 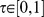 is the aging factor that allows trust to fade over time. If trust evaluation and uncertainty measure of node *i* on its neighboring node *j* need to be updated, Eqs. (10)∼(12) can be substituted into Eqs. (8)∼(9) for new results.

However, if indirect trust is integrated blindly with direct trust, the accuracy of trust evaluation is lowered because the neighboring nodes may be compromised to become malicious nodes, and provide false indirect trust information to deliberately exaggerate other malicious nodes or slander honest nodes. Therefore, indirect trust information should be given a certain weight to defend against false evaluation from malicious neighbors. 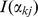, 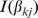 and 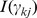 can be calculated in Eqs. (13)∼(15).
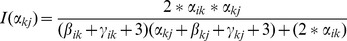
(13)

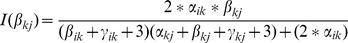
(14)

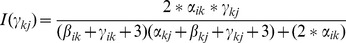
(15)


### Routing selection based on trust

In GEAR, the estimated cost and the learned cost are established for routing selection, and more details can be found in literature [6]. In TBDM-GR, 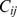 is used to represent the estimated cost or the learned cost calculated by node i about its neighboring node *j*. The newly proposed routing selection is based on not only the cost 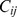 but also the trust evaluation 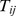, which is introduced in Section 3.3. The following section describes how to perform routing selection using cost and trust in order to enhance the robustness of GEAR.

Firstly, exclude some neighboring nodes whose trust values are lower than the average value of all neighboring nodes in the view of node *i*, and preprocess cost and trust of the remaining neighboring nodes. Cost and trust are two different attributes for node *i,* because cost is one of cost-based indicators which is the bigger the better and trust is one of efficiency indicators which is the smaller the better. The more excellent cost and trust, the bigger the processed values should be. Therefore, it is effective to select the next hop whose cost and trust are both better. The processed cost value and trust value of node *i* about its neighboring nodes are respectively denoted as 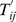 and 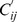 in Eqs. (16)∼(17).
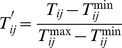
(16)

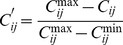
(17)


Where 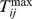 and 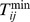 respectively represent the maximum and minimum trust values, and 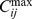 and 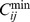 respectively represent the maximum and minimum cost values.

Secondly, 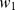 and 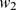 are introduced as weights of trust and cost respectively in order to reflect the difference of their importance in routing selection. 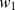 and 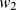 are determined by the eigenvector method [22] in Eqs. (18)∼(20).
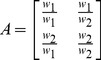
(18)

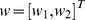
(19)

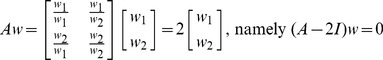
(20)


Where 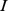 is the identity matrix, and the ratio of 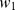 and 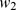 represents the relative importance of trust and cost. If the estimated values in matrix A are accurate, the left side of the equation is strictly equal to n-dimensional 0 vector. If the estimated values in matrix A are not accurate enough, small perturbation of elements in matrix A can lead to small perturbation of eigenvalues, hence the following Equation.
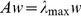
(21)


Where 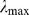 is the largest eigenvalue of the matrix A. Furthermore, the eigenvector, namely the weight vector 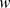 can be obtained by Eq. (21). In consideration of the data packets' importance and the non-malicious impact factor, namely the importance of the current routing task and non-malicious factors in the network, matrix A can be determined based on the relative importance table [23] provided by Saaty in 1980.

Finally, the three largest neighboring nodes of node *i* are chosen based on the calculated results of 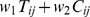. Data packets are forwarded to the node with the smallest uncertain measurement 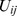 among the three nodes. Therefore, three candidate nodes are selected based on trust and cost, and the final next hop is selected based on the certain probability denoted as 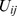. This not only reduces the impact of non-malicious factors to some extent, but also balances the routing load.

### Defending strategies based on trust

Three defending strategies against attacks in GEAR are provided to further enhance the protocol's robustness, as described in the following section.

#### Strategy 1. Dynamic initial trust

For node *i*, its neighboring node *j* is a reachable node through one-hop wireless transmission. At the beginning node *i* has no trust information about node *j*. Due to the introduction of trust in routing selection in GEAR, cost and trust are both considered. If node *i* sets trust values of all new neighboring nodes to 1, the trust mechanism becomes useless, and as a result malicious nodes can easily forge their own locations or claim to have the maximum energy level to launch attacks to GEAR. If node *i* set trust values of all new neighboring nodes to 0, this means that node *i* does not consider any new neighboring node as a reliable node, and this compromises the cooperation of new nodes and reduces the routing efficiency. In literature [14], the initial trust values of new neighboring nodes all have a fixed value of 0.5, which lacks flexibility and cannot well and truly reflect the initial trust state.

In our proposed trust-based defending model, the initial trust values of new neighboring nodes are dynamically set by the following policies. When node *i* finds a new neighboring node *j*, it randomly chooses three other neighboring nodes *l*, *m*, and *n* to inquire about the reliability of node *j* in the view of them. The feedbacks from node *l*, node *m*, node *n* about the reliability of node *j* are respectively denoted as 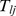, 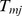 and 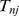. If there is no relevant information about node *j*, the feedback is 0. The initial trust value and uncertainty measure of node *i* on the new neighboring node *j* can be calculated in Eqs. (22)∼(23).
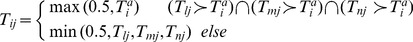
(22)

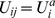
(23)


Where 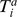 and 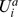 respectively represent the average values of node *i*’ trust values and uncertainty measures on its all neighboring nodes except node *j*. When the malicious node *j*, acting as a new neighboring node as described in Section 3.1, forges its own location and launches the Sybil attack, node *i* can dynamically obtain node *j*'s initial trust by inquiring its other neighboring nodes. This mechanism significantly reduces the opportunity of the new malicious node *j* with lower reliability to be selected as the next hop.

#### Strategy 2. Incentive mechanism

After launching the Sybil attack or claiming to have the maximum energy level and being selected as the next hop, the malicious node's continuous selective forwarding can lower reliability in the view of neighboring nodes, as calculated in Section 3.3. Afterwards the malicious node will be rarely selected as the next hop. However, forwarding requests from malicious nodes have not been restricted. In order to encourage cooperative behaviors of nodes, it is demanded that the higher reliability a node gets, the higher priority services it receives. If a node's reliability is low, its forwarding request may not be satisfied as expected. When a malicious node finds out that its request is denied or it is cleared out of the network, it will be forced to honestly forwarding data packets received from neighboring nodes to improve its reliability and meet its own request. For non-malicious nodes, honest forwarding behaviors bring them higher reliability, which in turn results in better routing services from neighboring nodes. Table 1 shows the relationship between reliability evaluation and available services that node *j* can obtain in the view of neighboring node *i*.

**Table 1:** Reliability evaluation and available services.

Reliability evaluation	Available services
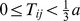	Clear the node out of the network.
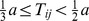	Does not respond to a forwarding request.
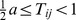	Respond to a forwarding request.

#### Strategy 3. Dealing with node mobility

Due to node mobility, a node may move out of the one-hop transmission range of another neighboring node, and this leads to the breakage of their neighborhood. When the neighboring node *j* moves out of node *i*'s one-hop transmission range, node *i* takes the following strategy in the beginning time 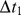. If 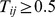, 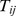 multiplied by the aging factor gradually becomes smaller and smaller until the value is equal to 0.5. Otherwise, 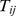 remains unchanged. In the next period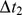 (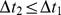), if node *j* does not recover its neighboring relationship with node *i*, node *i* will delete the information relevant to node *j*.

Similarly, a node may enter the one-hop transmission range of other nodes, and become one of their neighbors. If node *j* moves out of one-hop transmission range of node *i* and moves back within the time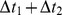, the value of 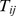 remains unchanged at the moment when node j enters. The remaining cases are in the scope of dealing with new joining nodes in the first strategy.

## Simulation Results and Analysis

We have performed simulation using OMNeT++ [24], a discrete event simulator. Original GEAR [6], CRATER [10] based on GEAR and the proposed defending model TBDM-GR in this paper are respectively implemented in simulation. The main simulation settings and parameters are provided in Table 2.

**Table 2:** Simulation settings and parameters.

Parameter	Value	Parameter	Value	Parameter	Value
Number of nodes	80	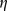 in GEAR	0.5	Communication rules	Random source to random destination
Network size	100*100 square units	Initial energy of a sensor node	500 units		
Transmission range	16units	Update interval of indirect trust information	10 s	Energy consumption	1 unit per sending
Network deployment	Random topology	Error probability caused by wireless channels and node malfunction	0.05		0.6 unit per reception
Average neighboring degree of a sensor node	12	Simulation duration	1000 s		Ignored in instruction processing

Each simulation is performed several times, and all the data used in the analysis is the average value of results in several experiments. We consider the following metrics for evaluating the performance of GEAR [6], CRATER [10] and TBDM-GR under multiple attacks: trust evolution, packet delivery ratio, and network lifetime. Trust evolution indicates the runtime trust value of a sensor node. Packet delivery ratio is defined as the percentage of sent packets actually received by the intended destinations. Network lifetime is measured by the first death of nodes in the network due to running out of energy.

### 

#### 1) Trust evolution

**Figure 1:**
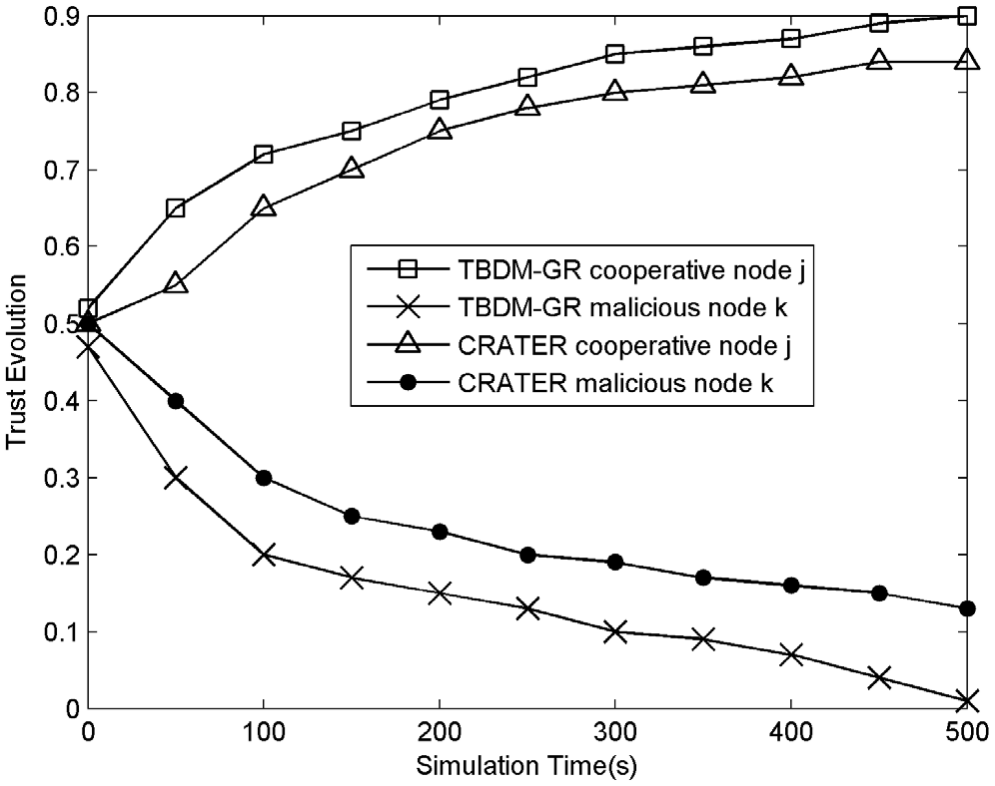
Trust evolution of a sensor node.

Node *i* has a non-malicious neighboring node *j* and a malicious neighboring node *k*. Node *j* honestly forwards data packets received from node *i*, while node *k* continuously refuses to forward data packets received from node *i*. In the view of node *i*, the reliability evaluation of the two neighboring nodes, namely trust evolution is shown in Figure 1. In original GEAR, trust mechanisms are not deployed so routing selection does not take into account the reliability of a node. In CRATER and TBDM-GR, the trust values of malicious nodes gradually decrease and the trust values of cooperative nodes gradually increase. Therefore, malicious attacks on routing selection are reduced while node *i* forwards data packets according to the reliability of neighboring nodes. However, compared to CRATER, TBDM-GR uses the Dirichlet distribution function for reliability evaluation and considers the importance of data packets being forwarded. According to Eqs. (3)∼(5), while cooperative nodes forwarding important data packets, their weighted number of successful forwarding times is counted more, so trust evaluation is more quickly increased; while malicious nodes refusing to forward important data packets, their weighted number of failed forwarding times is counted more, so trust evaluation is more quickly decreased.

#### 2) Packet delivery ratio

We change the percentage of malicious nodes in the network from 0% to 50% in steps, 5% each step. Malicious nodes forge their own locations or claim to have the maximum energy level. As soon as they are selected as the next hop, they refuse to forward the received data packets or drop 50% of the received data packets so as to implement selective forwarding. Meanwhile, according to the Byzantine Generals Problem [25], the percentage of malicious nodes in the simulation does not exceed 50%; otherwise the whole network is in an invalid state.

**Figure 2:**
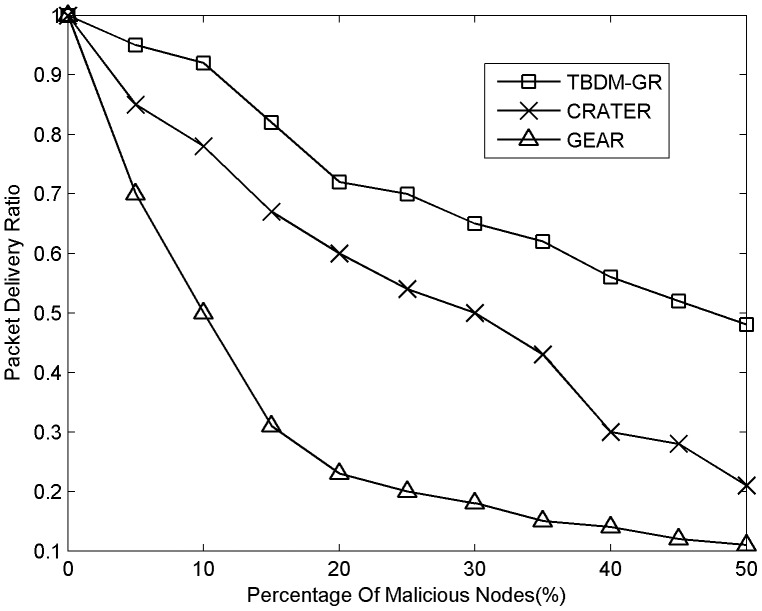
Packet delivery ration versus the percentage of malicious nodes.

As shown in Figure 2, the packet delivery ratio in TBDM-GR is much better than in CRATER and GEAR. Attacks from malicious nodes are not considered in the GEAR protocol, so data packets through malicious nodes will be discarded. In TBDM-GR trust evaluation are performed based on nodes' routing behaviors. With the multi-attribute decision-making method, the cost and trust of a node are synthetically considered in routing selection, which effectively reduces the possibility of malicious nodes being selected as the next hop. In addition, the dynamic initial trust, the incentive mechanism, and node mobility are also dealt with, which further encourages the cooperation of nodes. Therefore, TBDM-GR gets a better packet delivery ratio than CRATER. With the increase of proportion of malicious nodes, the successful forwarding rate of CRATER and GEAR are reduced to 20% and 10% respectively. The entire network is in an invalid state. While TBDM-G effectively resists malicious attacks, the successful forwarding rate decreases slowly and always maintains at 50% or more.

#### 3) Network lifetime

**Figure 3:**
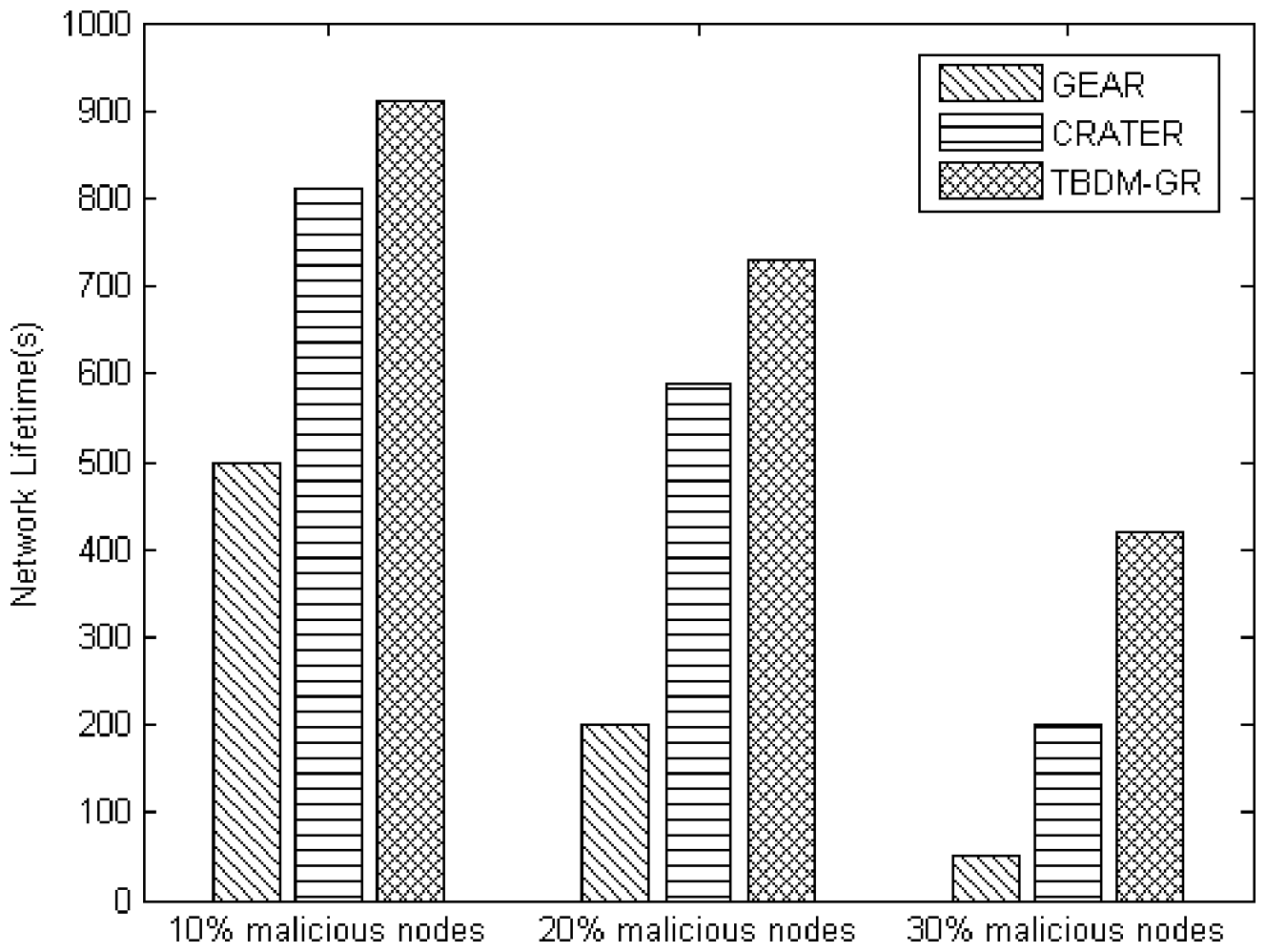
Network lifetime versus the percentage of malicious nodes.

Figure 3 shows a comparison of network lifetime in GEAR, CRATER and TBDM-GR with malicious node percentages of 10%, 20% and 30% respectively. TBDM-GR uses data packets' acknowledgements to evaluate node reliability instead of the more energy-consuming monitoring mechanism, and selects the next hop from reliable candidate nodes based the nodes' reliability and uncertainty to ensure successful delivery and balance routing load. In addition, the trust-based defending strategies in practice can promote the cooperation of nodes and reduce useless forwarding of data packets. Consequently, TBDM-GR has a longer network lifetime than GEAR and CRATER.

## Conclusion and Future Work

We proposed a trust-based defending model against multiple attacks in WSNs to enhance the robustness of GEAR. Trust evaluation is performed by means of the Dirichlet distribution, in which data packets' acknowledgements and importance are both used. Sensor nodes maintain trust for neighboring nodes in order to evaluate their reliability. Based on the multi-attribute decision-making method, the cost and trust of a node are synthetically considered in selecting the next hop on the routing path. In addition, trust-based defending strategies against attacks in GEAR are given, which effectively improve the packet delivery ratio and prolong the network lifetime. The future work mainly includes three aspects: firstly, introduce trust into other routing protocols in WSNs and adopt different strategies against attacks; secondly, further study the effectiveness of the trust mechanism and make use of trust to address data fusion, key management and other issues in WSNs; thirdly, in the actual scenarios, test and enhance the validity of the trust-based defending model.
